# Daily practice management of septate uterus: reproductive outcome after septoplasty

**DOI:** 10.52054/FVVO.13.3.032

**Published:** 2021-09-24

**Authors:** L Rousseau, G Brichant, M Timmermans, M Nisolle, L Tebache

**Affiliations:** Department of Obstetrics and Gynecology, University of Liège, CHR Liège, 4000 Liège, Belgium; Department of Obstetrics and Gynecology, Data Manager, University of Liège, CHR Liège, 4000 Liège, Belgium

**Keywords:** septate uterus, infertility, recurrent pregnancy loss, hysteroscopic septoplasty

## Abstract

**Background:**

Septate uterus is the most common uterine malformation found in women presenting poor reproductive history. Hysteroscopic septoplasty (HS) restores the uterine anatomy in a safe procedure.

**Objectives:**

The goal of our study was to determine the reproductive outcomes after HS of symptomatic septate uterus.

**Materials and Methods:**

In a retrospective observational single centre study the reproductive outcomes and complications after HS were evaluated in 31 women with symptomatic septate uterus. The patients were separated into two groups according to the symptoms - infertility or recurrent pregnancy loss (RPL).

Main outcome measures were the pregnancy and live birth rate and secondarily the complication rate. Furthermore, the results were analysed depending on the need of assisted reproductive techniques (ART).

**Results:**

The treatment resulted in an overall pregnancy rate of 71% for both groups. The spontaneous pregnancy rate was 45% and 8 pregnancies resulted from ART (26%). The overall first live birth rate is 51.6%. A decrease was seen in the miscarriage rate from 95.24% to 24% (p<0.001) in the overall population.

**Conclusions:**

In patients with a symptomatic septate uterus hysteroscopic septoplasty is a safe and effective procedure. The favourable results pointing out the benefits of surgery on the reproductive outcomes as well as the relatively simple and safe technique of HS make the intervention attractive.

## Introduction

Congenital uterine anomalies can be found in 5.5% of the general population, in 8% of infertile women and in 13.3% of women with a history of miscarriages (Chan et al., 2011). The true prevalence is probably underestimated because most affected women do not experience any reproductive problems and are therefore not always detected (Saravelos et al., 2008).

In up to 35% of the uterine anomalies, a septate uterus arising from incomplete resorption of the midline septum between the two Müllerian ducts is observed. The extent of the septum can vary between a slight midline septum to a complete septate uterus involving the endocervical canal. It is the most common congenital uterine anomaly identified in women with poor reproductive outcomes such as infertility, recurrent pregnancy loss (RPL) or preterm birth (Acien et al., 2004; Chan et al., 2011; Grimbizis et al., 2001; Mollo et al., 2009; Saravelos et al., 2008). Up to now, the mechanism for the impaired reproductive outcomes has not yet been established. The successful implantation of an embryo requires different conditions such as a normal morphology of the cavity, adequate uterine motility and a receptive endometrium (Bulletti and de Ziegler, 2006). These elements can be modified by malformations such as uterine septum, which is mainly composed of connective tissue and muscle fibers accompanied by vascularisation defects (Valle and Ekpo, 2013).

A symptomatic septate uterus can be surgically corrected. The gold standard treatment is hysteroscopic septoplasty. Several studies have indicated that it improves fertility and decreases the miscarriage rate with a low complication rate (Nouri et al., 2010, Saygili-Yilmaz et al., 2003), and some studies have pointed out that it is indicated before assisted reproductive techniques (ART) (Tomaževič et al., 2010).

Up until now, no consensus has been reached on the management of septate uteri, and it is still a great debate among the scientific community. Indeed, there is only one randomised controlled trial evaluating the impact of surgery on reproductive outcomes (Rikken et al., 2021). The randomised uterine septum transection trial – TRUST had some limitations such as the small amount of patients per centre, the various techniques used for the diagnosis and the open questions left with regard to the complete septum. Most other studies included patients with multifactorial aetiology of infertility, inducing selection bias, with a before/after design and were non-randomised comparative studies (Practice Committee of the American Society for Reproductive Medicine, 2016). We must also take into account the important heterogeneity in the definition of a uterine septum according to the classification system used (American Society for Reproductive Medicine-ASRM vs European Society of Reproduction and Embryology- ESHRE vs Congenital Uterine Malformations by Experts- CUME) (Ludwin and Ludwin, 2015). The concerns regarding the TRUST trial bring out the urgent need for more robust evidence in relation to the impact of hysteroscopic septoplasty.

This paper aims at analysing the reproductive outcomes in patients who presented with a symptomatic septate uterus and underwent a hysteroscopic septoplasty.

## Materials and methods

A retrospective search in our medical database provided 32 patients who received hysteroscopic septoplasty for a septate uterus at the University Department of Obstetrics and Gynecology CHU of Liège - site CHR Citadelle from July 2015 to December 2018 (Canadian Task Force classification II-2). The study was approved by the local ethics committee. Inclusion criteria were women with symptomatic septum from age 18 to 43 wishing to conceive. All types of septate uteri were included (U2a – incomplete septum n=24; U2b – complete septum n=7 - European Society of Human Reproduction and Embryology/ European Society for Gynaecological Endoscopy consensus on the classification of female genital tract congenital anomalies ESHRE/ESGE 2013) (Grimbizis et al., 2013). Patients with associated genital or pelvic diseases (myomas, endometriosis, adhesions and pelvic inflammatory disease) were excluded. A complete follow-up was available for 31 patients (96.87%).

The primary outcomes were the number of pregnancies and the number of first live births. We took a second pregnancy into account if the first ended in a miscarriage and defined an overall live birth rate.

The patients were evaluated in terms of their primary clinical problem and then subdivided into 2 groups. Group 1 was composed of 18 women (58%) with either primary infertility or secondary infertility with only one miscarriage. Infertility was defined as the inability to conceive after 12 months of regular unprotected sexual intercourse. Not all patients underwent ART before the septoplasty.

Group 2 consisted of 13 women (42%) who had experienced ≥ 2 consecutive miscarriages/ recurrent pregnancy losses (RPL) before 24 weeks of gestation. If RPL coexisted with infertility, the patient was included in the RPL-group. After the septoplasty they were subdivided according to the need of ART (Table I).

**Table I:** Characteristics of the patients and obstetric outcomes in infertility patients and patients with RPL.

		Group 1 : infertility	Group 2 : RPL	Total	p-value
N		18	13	31	
Median age (years) [range]		32.5 [24-40]	34 [28-40]	33 [24-40]	P=0.49
BMI (kg/m^2^) [range]		22 [19-29]	26 [19-32]	22 [19-32]	P=0.08
Duration of follow-up (months) [range]		19.5 [14-55]	16 [19-23]	18 [14-55]	P=0.89
Male infertility		5	3	8	
ART		11	6	17	
Complete septum		6	1	7	
Incomplete septum		12	12	24	
Pregnancies after HS (%)		12 (66.7%)	10 (77%)	22 (71%)	
	- Spontaneaous	7 (39%)	7 (54%)	14 (45%)	
	- ART	5 (27.7%)	3 (23%)	8 (26%)	
Median time to pregnancy (months) [range]		4 [2-39]	6 [1-30]	5.5 [1-39]	P=0.74
Miscarriages (%)		5 (35.71%)	1 (9%)	6 (24.13%)	
First live births (%)		7 (39%)	9 (69.23%)	16 ( 51.61%)	
Overall live births (%)		9 (50%)	10(76.92%)	19 (61.3%)	

We compared the pregnancy and live birth rates before and after the procedure. There was no statistical difference between the 2 groups regarding the age, the body mass index (BMI) and the duration of follow-up. We did not have a control group and the patients served as their own control. This is one restrictive point in the analysis.

The diagnosis of septate uterus was based on the ESHRE/ESGE consensus 2013 (Grimbizis et al., 2013). by using either magnetic resonance imaging (MRI) or three-dimensional ultrasonography (3D-US). The MRI was made by an expert pelvic radiologist. All ultrasound examinations were carried out by a gynecologist expert in ultrasonography assessment on an ultrasound system Samsung HS70A. The uterine septum was evaluated on a three-dimensional coronal view, and the residual myometrial thickness was measured to guide the surgeon during the procedure.

The hysteroscopic septoplasty was performed in the operating room either under general or under spinal anesthesia, using an operative hysteroscope fitted with a bipolar needle or a loop (TCRis 2.0 TransCervical Resection in Saline, Telescope 8.5 mm resectoscope, 4 mm optical lens, 12° direction of view, Tokyo, Japan, Olympus and a 4.3 mm Bettocchi hysteroscope, 2.9-mm optical lens, 30° direction of view, with 5 Fr. operating instruments, Karl Storz GmbH, Tuttlingen, Germany) with a saline infusion, with or without cervical dilatation. All procedures were executed by senior surgeons. The surgical procedure commenced with identifying both tubal ostia. The next step is the horizontal incision of the septum with the bipolar needle or loop equidistantly between the anterior and posterior uterine walls, starting from the lower part of the septum and continuing upwards to reach the uterine fundus, preserving 10-15 mm of the fundic myometrial wall. Three patients had a cervical septum, two of them were sectioned during the procedure.

Hyaluronic acid Hyalobarrier ® gel endo (Anika Therapeutics, Distributor Nordic Pharma) was injected inside the cavity immediately after the septoplasty in 39% of the patients (12/31) to prevent adhesions. The use of the gel was left to the appreciation of the surgeon without any further criteria. There was no postoperative use of an intrauterine device or hormone treatment. Most of the patients (87%, 27/31) had a diagnostic hysteroscopy within a month to evaluate the uterine cavity. The synechiae rate was 12.9% (4/31), and a remnant septum (>10mm) was observed in 9 patients (29%). A second intervention was needed in three patients for a remnant septum above 10 mm (9.6%), and the 6 others (19.35%) benefitted from an immediate resection by means of an office hysteroscope using scissors and a saline infusion.

The follow-up consisted of a search in our medical database evaluating the numbers of pregnancies, live births, miscarriages, spontaneous late abortions and any form of ART. Not every patient delivered in our centre, therefore we did not have the data about the birthweight, mode of delivery and gestational age of delivery. Consequently, we did not include this in our analysis.

Intrauterine pregnancy was characterised by the presence of an embryo with a heartbeat during a transvaginal ultrasound examination. Miscarriage was defined as pregnancy loss before 24 weeks of gestation. No routine cervical cerclage was performed. The delivery and obstetrics complications were noted in the database. Live birth was determined as the birth of a live baby after 24 completed weeks of gestation.

The statistics are expressed as median [range] for the continuous variables and as frequencies and percentages for the discrete data. Differences in reproductive outcomes before and after HS are evaluated using Chi-square. A p-value <0.05 is considered statistically significant. The statistics were performed with R software (Development Core Team (2005). R).

## Results

The median follow-up time was 25 months [14-55]. Patients’ median age at intervention was 33 years [24-40] in the overall population, 32.5 [24-40] in Group 1 and 34 [28-40] in Group 2, and the body mass index (calculated as weight in kilograms divided by the square of height in meters) was 22 [19-32] in the overall population (Group 1 : 21.5 [19-29], Group 2 : 24 [19-32]) (Table I).

The overall pregnancy rate (PR) after hysteroscopic septoplasty was 71 % (22/31) and the overall live birth rate was 61.3% (19/31). The first live birth rate (LBR) was 51.6% (16/31). Six patients experienced a miscarriage (19.35%), but three of them achieved a second pregnancy afterwards. The median time interval between surgery and pregnancy was 5.5 months [1-39] (Table I).

Furthermore, the spontaneous pregnancy rate was taken into account. In the overall population, 14 patients had a spontaneous pregnancy after hysteroscopic septoplasty (PR 45.16%). Seventeen patients had ART. Among them, 8 patients got pregnant (PR 26%) (one after 1 attempt at in vitro fertilisation [IVF], 5 after 2 intracytoplasmic sperm injections, and 2 after 3 attempts at IVF) and 5 achieved a live birth (LBR 16.13%).

### Group 1: infertility

Twelve out of 18 patients got pregnant (PR 66.7%). Five of the pregnant women had a miscarriage. The first live birth rate was 39% (7/18). Among these 5 patients, 2 had a new pregnancy resulting in 2 live births. This gives us a total number of pregnancies of 14. The overall live birth rate was 50% (9/18). The median time interval between surgery and pregnancy was 4 months [2-39]. Five of the 12 pregnancies resulted from ART (41.6%) (Table I).

### Group 2 : recurrent pregnancy loss

The overall pregnancy rate was 76.92% (10/13), and the first live birth rate was 69.2% (9/13). One patient experienced a miscarriage, but achieved a live birth afterwards. The overall live birth rate was 76.92% (10/13). The median time interval between surgery and pregnancy was 6 months [1-30]. Three of the 10 pregnancies resulted from ART (30%) ( Table I).

### Comparison of preoperative and postoperative data

Before the treatment by hysteroscopic septoplasty, there was a total number of 63 pregnancies in the overall population from which 60 ended in miscarriage. After the hysteroscopic septoplasty, 25 pregnancies were counted from which 6 ended in miscarriage. This indicates a decrease in the miscarriage rate from 95.24% to 24%, which is statistically significant (p <0.001). The live birth rate increased from 9.6% (3/31) to 61.3% (19/31) (p<0.001) (Table II).

**Table II:** Comparison of preoperative and postoperative live birth rates and miscarriages in Group 1 and Group 2.

		Before septoplasty	After septoplasty	p-value
Overall population (n=31)				
	Total number of pregnancies*	63	25	
	First live births (%)	3 (9.6%)	16 (51.61%)	<0.001
	Overall live births (%)		19 (61.3%)	<0.001
	Miscarriages (%)	60 (95.24%)	6 (24%)	<0.001
Group 1 : infertility (n=18)				
	Total number of pregnancies*	5	14	
	Cumulative live births (%)	0	9 (50%)	<0.001
	Miscarriages (%)	5 (100%)	5 (35.71% )	<0.02
Group 2 : RPL (n=13)				
	Total number of pregnancies*	58	11	
	Cumulative live births (%)	3 (23.07%)	10 (76.92%)	<0.01
	Miscarriages (%)	55 (94.82%)	1 (9.1%)	<0.001

* total number of pregnancies : the second pregnancy was taken into account if the first ended in a miscarriage

The drop in miscarriages was even more impressive in the second group with a decrease from 94.82% to 9.1% (p <0.001). In the Infertility-group, the live birth rate increased from 0% to 50% (p <0.001) (Table II).

Our complication rate was 3.2% (1/31). No intraoperative complications were noticed. We had one late complication consisting in a uterine perforation following a postpartum IUD insertion. There were no preterm births.

## Discussion

This retrospective study intended to observe the reproductive outcomes of women presenting with a septate uterus associated with infertility or pregnancy loss who underwent hysteroscopic septoplasty. In the manuscript, we demonstrated the benefits of hysteroscopic septoplasty in terms of pregnancy rate and live birth rate obtained after surgery either spontaneously or after ART.

Uterine septum accounts for 35% of the uterine anomalies and is associated with the highest rate of adverse reproductive outcomes (Homer et al., 2000). The prevalence of congenital uterine anomalies in women with reproductive failure remains unclear, largely due to methodological bias. These anomalies can remain asymptomatic and are thus not always detected. There is an important heterogenicity in the diagnostic methods, and finally the classification systems vary. Nevertheless, congenital uterine anomalies have been clearly linked to women suffering from recurrent pregnancy loss (Acien, 1993; Grimbizis et al., 2001; Raga et al., 1997; Venetis et al., 2014). Saravelos et al. (2008) stated in a review that there was also an association between a septate uterus and infertility.

Indeed, the current data suggest a better reproductive outcome and an improvement in the pregnancy rate after hysteroscopic septoplasty (Grimbizis et al., 2001; Homer et al., 2000; Mollo et al., 2009; Pabuccu et al., 2020; Tomaževič et al., 2010). A systematic review by Nouri et al. (2010) of 18 different studies with a total number of 1501 women showed an overall pregnancy rate of 60.1% (592/1501) and a live birth rate of 45% after intervention (686/1501). In our study, the pregnancy and the live birth rates after hysteroscopic septoplasty were similar to the results discovered in the literature (Group 1 PR 66.67%, LBR 50%; Group 2 PR and LBR 77%). Pabuccu et al. (2020) conducted a prospective observational study on patients with a septate uterus, unexplained infertility and repeated IVF/ ICSI failure. After the surgery 46% with a septate uterus conceived spontaneously. The mean time to pregnancy was 8.7 months ±1.6. Our spontaneous pregnancy rate was 45.16%. The mean time to pregnancy was 5.5 months. In a retrospective case study including 361 patients with septate uterus, Saygili-Yilmaz et al. (2003) reported a decrease in the miscarriage rate from 96.1% to 10.2% and a raised overall live birth rate from 2.4% to 75% in the RPL-group after the intervention. In our study, comparable data relating to the miscarriage rate were noticed with a decrease from 95.24% to 24% in the overall population (p< 0.001) and even an impressive drop from 94.82% to 9,1% in the RPL group (p<0.001). Similarly, the live birth rate increased from 9.6% (3/31) to 61.3% (19/31) in the overall population (p<0.001).

On the other hand, there are some comparative studies that found no significant difference between hysteroscopic septoplasty and expectant management in women with symptomatic septate uterus (Heinonen, 1997; Kirk et al., 1993; Rikken et al., 2020). But like many other studies, these ones are also prone to biases as discussed in a paper by Grimbizis et al. (2020). They pointed out the unexpected recommendation based on the retrospective study from Rikken et al. They urged surgeons not to change their practice and to offer hysteroscopic septoplasty to patients suffering from a symptomatic septate uterus.

Thus, hysteroscopic septoplasty remains largely debated since there have not been any solid randomised controlled trial on the effectiveness of a septum resection so far. The Randomised Uterine Septum Trial (TRUST Trial)7 found no statistically significant difference between resection and expectant management in women with RPL or subfertility and an incomplete septate uterus (Rikken et al., 2021). This study was criticised by the scientific community, and some bias has been identified. First, most patients had an incomplete septum. There was also no answer regarding the surgical removal of a complete uterine septum. Other questions relating to the outcomes concerning pregnancy loss, ongoing pregnancy, preterm birth and fetal malposition remain open. Also, the septum was diagnosed using various techniques (MRI, US-3D, HSG, hysteroscopy associated with laparoscopy). It was defined as a multicentric study, but most centres had less than 10 cases in an eighteen-year period. Another critical point was the inclusion criteria changed twice during the study period. And finally, only 69% of the patients had a postoperative follow-up by hysteroscopy.

A crucial critical point is the classification system used to define a septate uterus. In order to compare the results of surgical treatment and expectant management, a worldwide definition of a septate uterus is needed. Ludwin compared the ESHRE/ESGE 2013 (U2a – incomplete septum; U2b – complete septum) and the ASRM systems (American Society of Reproductive Medicine type Va – complete septum and Vb – incomplete septum) (Ludwin and Ludwin, 2015).. In their study of 261 patients, the ESHRE/ESGE system diagnosed more numbers of septate uteri than the ASRM system (80 vs 12 out of 261). A septate uterus defined by the ESHRE/ESGE system mainly corresponded to an arcuate uterus for the ASRM classification Consequently, according to the results of Ludwin, the prevalence of septate uterus is overestimated by the ESHRE/ESGE classification and therefore overtreated, but underestimated by the ASRM leaving a “grey-zone”. Ludwin et al. (2019) urged the experts to review the current classification system in order to prevent “overdefinition, overdiagnosis and overselling” of septate uteri. This critical point was also emphasised by Grimbizis et al. (2020).

There is no consensus relating to the size, length and width of the uterine septum to treat because there are only few small studies about this subject. Fedele et al. compared 23 patients with a complete uterine septum to 79 patients with an incomplete septum (1993). After the surgery, no significant difference on the live birth rate was observed (75% (17/23) vs 67% (53/79), p=0.5). Current data suggest that the composition of the septum differs between patients with RPL and infertility regarding the amount of muscle fibers and vascularisation. Both are more important in patients with RPL whereas patients with infertility have a septum with more connective tissue. This could be explained by the underlying embryological defects leading to their formation (Fascilla et al., 2019; Grimbizis, 2019).

Regarding hysteroscopic septoplasty before ART, Tomaževič et al. (2010) conducted a retrospective matched control study of patients (n = 2 481) divided into 3 groups : septate uteri before hysteroscopic septoplasty (n=289), septate uteri after intervention (n=538) and patients without any malformation (n=1654). They all underwent IVF/ICSI with an embryo transfer. The first group, patients before surgery, showed significantly lower pregnancy and live birth rates compared to normal uteri (12.4% vs 29.2%, p<0.001; 2.7% vs 21.7%, p<0.001). The miscarriage rate was also significantly higher compared to control (77.1% vs 16.7%, p< 0.001). After the resection, the results were comparable to normal uteri (pregnancy rate : 22.9% vs 26%, live birth rate 15.6% vs 20.9%, miscarriage rate : 29.2% vs 18.4%). It shows that hysteroscopic septoplasty improves the implantation rate. Therefore, prior to any ART treatment, the patients should benefit from it. In our study, seventeen patients had ART after HS. Among them, 8 patients got pregnant (PR 26%).

Hysteroscopic septoplasty is a safe procedure used in a routine basis by most gynaecologists. According to a pooled analysis by Nouri and colleagues of 15 studies enrolling 1324 women who underwent hysteroscopic septoplasty, a complication rate of 1.7% was determined (23/1324), mostly due to perforations (15/1324 = 1.1%) (Nouri et al., 2010). In our study, a complication rate of 3.2% (1/31) was noticed due to the migration of a Copper-IUD through the uterine scar after a postpartum insertion. No study showed any significant increase of uterine rupture (Practice Committee of the American Society for Reproductive Medicine, 2016), and only case reports could be found in the literature. The main risk factors identified were excessive septal incision, penetration of the myometrium, uterine wall perforation and excessive use of cautery during the procedure (Valle and Ekpo, 2013). Furthermore, the surgeries were carried out by several senior surgeons, thus implying different surgical techniques and the use of various devices. Budden reviewed the literature and identified 11 studies assessing the impact of surgical techniques. They found no evidence in favour of one particular technique, but they emphasised that the technique and the device used should be those the surgeon feels most comfortable with (Budden and Abbott, 2018). By using the adequate technique and the right tool, hysteroscopic septoplasty seems to be a simple, effective and safe procedure.

But these studies only represent observational trials without any prospective randomisation (Practice Committee of the American Society for Reproductive Medicine, 2016). Our study happened to induce the same methodological bias. It must be clearly stated that the statistical power of the current study is low. It was an observational retrospective study with a before/after design where the patients served as their own controls. In fact, for ethical reasons, we felt unable to build up a control group while leaving patients with a septum, who were willing to conceive and who suffered from infertility or RPL, without any treatment or with a delayed treatment. Besides, we were confronted with an important bias of selection due to the small number of patients, their important reproductive history, the heterogeneity of the groups, the small amount of complete septum and additionally the male infertility for some couples. Also, the assessment of the uterine cavity was based on varied diagnostic methods. Finally, after the procedure, the patients attempted conception without any strict protocol, some more motivated than others.

The heterogeneity in management reflects the reality of the work as far as the treatment of septate uterus is concerned. Thus, hysteroscopic septoplasty remains largely debated. The level of evidence by the TRUST is a level 2 ( RCT < 100 patients), and compared to the positive experience accumulated in literature (mostly observational trials level 3), the favourable results pointing out the benefits of surgery on the reproductive outcomes as well as the relatively simple and safe technique of hysteroscopic septoplasty make the intervention attractive for the patient. The TRUST does not propose any surgery to patients with a symptomatic septate uterus, but this recommendation is based on just one study. A well- designed and well-conducted study, based on a strict diagnosis and classification system with a standard surgery, a standard use of tools and a standard post- operative management is still needed.

## Conclusion

Hysteroscopic septoplasty in patients with a septate uterus and adverse reproductive outcomes is recognised as a safe and simple procedure with an overall live birth rate of 61.3%, which this study highlighting an increase from 9.6% to 61.3% (p <0.001) following treatment. Also, a statistically significant reduction in the miscarriage rate, mainly in the RPL group (from 94.82% to 9.1%; p<0.001), was observed.
